# ASCO 2020 non-small lung cancer (NSCLC) personal highlights

**DOI:** 10.1007/s12254-020-00673-2

**Published:** 2021-01-13

**Authors:** Lena Horvath, Andreas Pircher

**Affiliations:** Department of Hematology and Oncology, Internal Medicine V, Medical University of Innsbruck, Anichstraße 35, 6020 Innsbruck, Austria

**Keywords:** EGFR, Osimertinib, Ipilimumab, Nivolumab, Atezolizumab, Tiragolumab, Trastuzumab deruxtecan

## Abstract

In this article we summarize our personal non-small cell lung cancer (NSCLC) highlights of the virtual ASCO 2020 meeting, covering developments in early and advanced-stage NSCLC. Until recently early stage NSCLC patients were treated independently of their genetic profile. Now the ADAURA study proved that postoperative osimertinib significantly prolongs disease-free survival compared to standard chemotherapy in EGFR-mutated NSCLC , underlining the high efficacy of targeted therapies in early stages. In advanced-stage disease, of course immunotherapy (IO) was at the center of attention. Final analysis of KEYNOTE-189 (pembrolizumab plus chemotherapy versus chemotherapy alone) and 3‑year update of CheckMate 227 (nivolumab plus ipilimumab versus standard chemotherapy) proved the long-term overall survival benefit of IO combinations in the first-line setting independent of PD-L1 status. The innovative CheckMate 9LA study demonstrated rapid disease control with limited-course chemotherapy plus IO doublet, while sparing chemotoxicity and may soon become a new clinical treatment choice. Moreover, the phase II CITYSCAPE trial presented significant response rates of the TIGIT-inhibitor tiragolumab plus atezolizumab in PD-L1 positive NSCLC. For HER2-mutated patients a highly effective drug conjugate trastuzumab deruxtecan was presented in a phase II study, extending targeted agents in genetically driven NSCLC. Altogether, ASCO 2020 could excite with inspiring new data for an optimized and more individualized NSCLC treatment regimen, contributing to a better outcome for both early and late-stage diseased patients and continuing to decrease lung cancer mortality.

## Introduction

The 56th annual ASCO 2020 meeting was held entirely virtual under the theme “Unite and Conquer: Accelerating Progress Together”, appropriately addressing both the great amount of new research presented as well as the challenging time of the COVID-19 pandemic.

Again, treatment of non-small cell lung cancer (NSCLC) was a major aspect of the meeting. In advanced-stage disease, long-term OS results from KEYNOTE-189 and CheckMate 227 once more underpinned the impressive clinical relevance of immunotherapy (IO) in the first-line setting [2, 3]. The number of novel IO combinational approaches to fight the evolution of IO resistance are growing and broaden the spectrum of therapeutic options. With highly effective and well-tolerated targeted therapies available, the importance of genetic profiling in both early and advanced-stages has now become apparent. Despite rapid improvements, a personalized therapy stratification is still lacking and biomarkers beyond PD-L1 remain uncertain. Discussing the right treatment sequence will be increasingly important to further improve patient care.

## Early stage non-small cell lung cancer

Postoperative pathological staging defines whether adjuvant chemotherapy should be offered to the patients or not [7]. At ASCO 2020, the first randomized phase III data were presented on targeted therapies in the adjuvant setting for EGFR-mutated NSCLC in stages IB–IIIA [1].

### ADAURA

The ADAURA study is a double-blind, randomized phase III study evaluating the efficacy and safety of adjuvant osimertinib (a CNS active third-generation EGFR tyrosine kinase inhibitor) vs. placebo in 682 patients with EGFR-mutated (del19/L858R) stage IB–IIIA NSCLC after complete tumor resection, for a maximum of 3 years. Postoperative chemotherapy for 4 cycles was allowed. The primary study endpoint was disease-free survival (DFS) of patients in stages II/IIIA. Secondary endpoints were overall survival (OS), DFS in stages IB–IIIA, and safety. Due to the effectiveness of adjuvant osimertinib, the study was unblinded prematurely in April 2020 upon recommendation by the Independent Data Monitoring Committee (IDMC) and results of the unplanned interim analysis were presented at ASCO 2020 and recently published in the *New England Journal of Medicine* [1]. The study met its primary endpoint and showed that osimertinib significantly prolongs DFS (in the osimertinib arm not yet reached) compared to placebo (20.4 months; hazard ratio [HR] 0.17, *p* < 0.0001), corresponding to a reduction in the risk of recurrence by an impressive 83%. The inclusion of stage IB patients (secondary endpoint) did not significantly alter the DFS prolongation by osimertinib (placebo vs. osimertinib: DFS 28.1 months vs. not achieved; HR 0.21, *p* < 0.0001). The DFS benefit with osimertinib was observed irrespective of whether patients received adjuvant chemotherapy or not. Furthermore, patients who received osimertinib had fewer locoregional and distant relapses and fewer CNS recurrence events than those who received placebo (1% vs. 10%). Regarding adverse events (AE), osimertinib was well tolerated, as previously experienced in the treatment of advanced stages.

As data from the interim analysis was still premature at time of presentation, a longer follow-up should be warranted before setting a new therapy standard. Furthermore, DFS data does not necessarily translate into a longer OS, as demonstrated by the example of the CTONG1104 study investigating gefitinib (first-generation EGFR-TKI) as adjuvant therapy for 2 years vs. 4 cycles conventional adjuvant chemotherapy in stage II–IIIA completely resected NSCLC with EGFR mutation [8]. Although the median DFS was significantly longer under gefitinib than under chemotherapy (28.7 vs. 18.0 months; HR 0.60, *p* = 0.005; median follow-up: 36.5 months), no OS benefit was achieved after a median follow-up of 76.9 months (median OS: 75.5 vs. 79.2 months, *p* = 0.823). At the moment the use of adjuvant chemotherapy depending on the pathological stage remains standard.

## Targeted therapies in genetically driven NSCLC

### HER2 mutation as new target

Developments were also reported on personalized NSCLC therapy and the HER2 mutation was identified as a promising treatment target. The DestinyLung01 study showed that the antibody-drug conjugate (ADC) trastuzumab deruxtecan (T-DXd) led to a confirmed objective response rate (ORR) of 61.9% in patients with relapsed/refractory HER2-mutant NSCLC (*n* = 42) [6]. At a median follow-up of 8 months, the median PFS was 14.0 months. The median duration of response (DOR) and the median OS had not been reached. Serious treatment-emergent adverse effects (TEAE) were reported in 16.7% of patients. The most common TEAEs leading to dose interruption were decreased neutrophil count (19.0%) and lung infection (7.1%). In summary the data are very promising and also comparable with efficacy in other genetic alterations. Furthermore, based on these data the FDA granted a breakthrough therapy designation to T-DXd for the treatment of patients with metastatic NSCLC whose tumors have a HER2 mutation and disease progression on or after platinum-based therapy. The evaluation of efficacy of T-DXd on HER2^high^ expressing patients is ongoing.

## Advanced stage non-small cell lung cancer

### IO combination therapy

The use of IO has become our clinical standard in locally advanced and metastatic NSCLC patients, as monotherapy or in combination with chemotherapy depending on PD-L1 status and disease burden. Impressive long-term OS and PFS results were updated at ASCO 2020 with the final analysis of KEYNOTE-189 [3] (pembrolizumab plus chemotherapy) as well as with the 3‑year update of CheckMate 227 [2] (nivolumab plus ipilimumab vs chemotherapy alone), both independent of PD-L1 status. However, IO resistance still depicts a major hurdle in the majority of patients with disease progression (primary versus secondary resistance). At the ASCO 2020 meeting, the trials CheckMate 9LA [4] and CITYSCAPE [5] presented innovative first-line IO combination regimens to further improve IO responses in advanced stage NSCLC patients.

### CheckMate 9LA

The phase III CheckMate 9LA study investigated frontline nivolumab/ipilimumab plus the addition of a limited-course chemotherapy (2 cycles platinum doublet) vs. 4 cycles chemotherapy alone (with optional pemetrexed maintenance). After a minimum follow-up of 12.5 months the study showed a significantly longer median OS (15.6 vs 10.9 months; HR 0.66,), median PFS (6.7 vs 5.0 months; HR, 0.68) and median DOR (11.3 vs 5.6 months) with the combination therapy compared to chemotherapy alone. The observed OS benefit was independent of histology, PD-L1 status and absence/presence of brain metastasis. Patients older than 75 years and never-smokers did not benefit. Toxicity rates were moderately higher in the combination therapy (47% vs 38%), while chemotherapy-related AE were lower with the limited course. Altogether, CheckMate 9LA presented an interesting treatment approach by combining rapid disease responses expected from chemotherapy with long-term IO benefits. However, the advantage of double IO in this setting remains uncertain and longer follow-up data will be needed before implementation into clinical practice.

### CITYSCAPE

In addition to well-known PD‑1 and CTLA‑4, alternative immune checkpoints are constantly being discovered and evaluated for the utility as druggable targets. The phase II CITYSCAPE trial investigated the novel anti-TIGIT antibody tiragolumab in combination with PD-L1 inhibitor atezolizumab vs. placebo plus atezolizumab in 135 patients with stage IV PD-L1 positive NSCLC. Patients were stratified by PD-L1 expression, histology and tobacco history. After a median follow-up of 10.5 months the study could show a significant ORR improvement for the combination (37% vs. 21%) as well as PFS improvement (5.6 vs. 3.9 months; HR 0.58), while toxicity was not aggravated. The authors underlined the particular benefit of additional tiragolumab in PD-L1^high^ expressing patients (TPS > 50%), with a respective ORR of 66% compared to 24% with atezolizumab monotherapy. In response to these results, atezolizumab/tiragolumab will now be further evaluated in the phase III study SKYSCRAPER-01.

#### Take home message

**Osimertinib** is the first adjuvant targeted therapy to show a significant and clinically relevant advantage in disease-free survival in EGFR-mutated non-small cell lung cancer (NSCLC) after complete tumor resection.

**Trastuzumab deruxtecan** shows promising clinical efficacy in HER2-mutated NSCLC with acceptable toxicity profile.

**Nivolumab/Ipilimumab** combined with 2 cycles of standard chemotherapy is a highly effective combination therapy in the first-line setting of metastatic NSCLC.

**Tiragolumab** **+** **atezolizumab** is effective and safe in the first-line setting of advanced NSCLC with particular benefit in PD-L1^high^ expressing patients.
